# Overestimating the Effects of Healthy Aging

**DOI:** 10.3389/fnagi.2015.00164

**Published:** 2015-08-26

**Authors:** Vanja Kljajevic

**Affiliations:** ^1^University of the Basque Country (UPV/EHU), Vitoria & IKERBASQUE, Basque Foundation for Science, Bilbao, Spain

**Keywords:** cognitive aging, Alzheimer’s disease, biomarkers, amyloidosis, neurodegeneration, neuroimaging

Typical cognitive aging is often defined as aging free of dementia, and yet it does not seem to exclude dementia-related pathology. The most common type of dementia is Alzheimer’s disease (AD) – it is a progressive neurodegenerative disease that affects multiple cognitive domains, including memory, attention, reasoning, judgment, language, as well as behavior. AD accounts for 60–80% of dementia cases, currently affecting 35 million people worldwide and progressing toward an estimated 115 million by the year 2050 (Riedel, 2014). Since there is no cure for AD, the research focus has shifted from the symptomatic stage to the earlier stages of the disease, with the goal to develop treatments that would effectively delay the onset of symptoms and progression of this devastating disease (Selkoe, 2012; Bateman, 2015). The need to diagnose and begin treatment even before overt symptoms appear has generated in neurological researchers a strong interest in cognitively healthy people who are at risk for AD. Recent modifications of the research and diagnostic criteria for AD reflect this trend by including cognitively normal (CN) at-risk-for-AD persons as a preclinical stage in the AD continuum (Dubois et al., 2007, 2010; Sperling et al., 2011).

Crucial in the modified criteria for AD is the concept of biomarkers of Alzheimer’s pathology, i.e., physiological and anatomical parameters that can be objectively measured to establish the presence of changes due to the disease (Jack and Holtzman, 2013). Since amyloid-β neuritic plaques and neurofibrillary tangles have been established as the pathological hallmarks of AD, biomarkers of amyloidosis and neurodegeneration are used as *in vivo* indicators of the presence of Alzheimer’s pathology (Dubois et al., 2007, 2010; Sperling et al., 2011; Jack and Holtzman, 2013). The most validated AD biomarkers are cerebrospinal fluid (CSF) amyloid-β_1–42_, total tau and phosphorylated tau as pathophysiological biomarkers of Alzheimer’s pathology, and brain regional structural and metabolic changes, as topographical biomarkers (Dubois et al., 2007; Jack and Holtzman, 2013). However, it has been recently recognized that the topographical markers lack pathological specificity necessary for diagnosis, and that they are more useful for measuring the disease progression (Dubois et al., 2014). According to this view, *in vivo* evidence of Alzheimer’s pathology, and thus preclinical AD stage, would constitute increased cerebral amyloid burden/decreased CSF amyloid level together with increased levels of total tau and phospho-tau. The general idea is that if a person with Alzheimer’s pathology lives long enough, they will eventually progress to AD dementia. Thus, biomarkers-based diagnosis provides an opportunity to introduce early treatments that will attempt to delay appearance of dementia symptoms and disease development.

While the value of early diagnosis is beyond any dispute, the question of how to disentangle typical aging and preclinical AD remains open. This issue is further complicated by the heterogeneous nature of the preclinical AD. According to one model, preclinical AD comprises three stages: at stage 1, only brain amyloidosis is evident; at stage 2, both cerebral amyloidosis and neurodegeneration are present; and at stage 3, these features are further combined with subtle cognitive changes (Sperling et al., 2011). Two additional stages have been added to this model: stage 0, which represents cognitively intact people without brain amyloidosis, neurodegeneration, or subtle cognitive changes, and suspected non-amyloid pathology (SNAP), which is characterized by normal amyloid markers and abnormal neurodegeneration markers and which is not necessarily related to AD (Jack et al., 2012). While it is clear that preclinical AD is characterized by the absence of cognitive impairment on objective measures of cognition, subtle cognitive changes that have been associated with this stage of disease remain poorly understood (Dubois et al., 2014).

Another model of preclinical AD has recently been proposed in the context of a cross-sectional study that assessed a population-based sample of 985 CN people aged 50–89. The model combines only amyloidosis [positive (A^+^) or negative (A^−^)] and neurodegeneration [positive (N^+^) or negative (N^−^)] status, leaving out subtle cognitive changes. The study revealed that the estimated population frequency of the A^−^N^−^ sequence was 100% at age 50, but only 17% at age 89, with the A^+^N^+^ sequence reaching 42% by age of 89, whereas the frequency of the sequence A^−^N^+^ was 24% by that age (Jack et al., 2014). Examples of pathological sequences leading to AD dementia are as follows: A^−^N^−^ to A^+^N^−^ to A^+^N^+^, and A^−^N^−^ to A^−^N^+^ to A^+^N^+^, whereas the two component sequence A^−^N^−^ to A^−^N^+^ is linked to heterogeneous underlying pathology (Jack et al., 2014). However, the neurodegenerative status in this study was determined based on topographical biomarkers of AD, more specifically by an AD signature ^18^F-fluorodeoxyglucose (^18^F-FDG) PET and hippocampal volume on MRI. These topographical markers are not unique markers of Alzheimer’s pathology; for example, hippocampal volume is reduced in other conditions, such as frontotemporal dementia, hippocampal sclerosis, Lewy-related pathology, argyrophilic grain disease, diabetes, and bipolar disorder, among others (Jack et al., 2012; Dubois et al., 2014). Thus, although these findings still suggest that pathological aging is a predominant way of cognitive aging, they also indicate a need for consensus on which biomarkers are suggestive of Alzheimer’s pathology only and thus better suited for untangling preclinical AD and the healthy brain aging.

As models of preclinical AD continue to develop, a challenge to the field is to reconcile the evidence of AD-related pathology found in a large number of CN elderly people (Jack et al., 2012, 2014) with the notion of “healthy” or “successful” aging (Rowe and Kahn, 1987). This evidence seems to question the research practice of not considering possible presence of Alzheimer’s pathology in CN elderly participants when including healthy elderly persons in cognitive studies. However, without the actual evidence to exclude Alzheimer’s pathology, one can assume that some percentage of CN elderly subjects in such studies may represent preclinical AD. This problem has been occasionally recognized (Gold et al., 2013; Brier et al., 2014). It clearly requires a systematic change in approach, because subtle cognitive changes, reliance on cognitive strategies, and networks’ reorganization that one would interpret as the effects of healthy aging might actually reflect the disease progression. While the number of studies investigating that the impact of atrophy, hypometabolism, white matter changes, and ApoE4 on cognitive processes across the AD stages is consistently growing, possible effects of β-amyloid, t-tau, and p-tau on cognitive processes in preclinical AD remain largely unexplored (Riedel, 2014).

Finally, in addition to apparently small percentage of CN persons who despite an advanced age resist Alzheimer’s pathology (Jack et al., 2012, 2014), there exist so-called SuperAgers. These are elderly people (80+) who appear to have healthy brains and well-preserved memory abilities. A recent study involving 12 SuperAgers found that their memory abilities were comparable to those of a group of healthy 50- and 65-year-old persons. When compared to a group of healthy age-matched peers on measures of cortical thickness, the SuperAgers had significantly thicker cerebral cortex. Furthermore, the left anterior cingulate was significantly thicker in SuperAgers compared to both groups (Harrison et al., 2012). Thus, we find not only neuropathology but also healthy brains and preserved memory at well-advanced age.

In conclusion, it is now possible to establish the presence/absence of Alzheimer’s pathology *in vivo* by measuring parameters that indicate biological changes caused by AD, thereby determining if a person is at risk for developing AD. Incorporating such evidence into cognitive aging research allows a differentiation of possible influences of Alzheimer’s pathology from the effects of healthy aging on cognitive processes. Only by systematically incorporating such evidence in research on cognitive aging, we will be able to make a progress in disentangling preclinical AD from healthy cognitive aging.

## Conflict of Interest Statement

The author declares that the research was conducted in the absence of any commercial or financial relationships that could be construed as a potential conflict of interest.
